# Long-term dataset on aquatic responses to concurrent climate change and recovery from acidification

**DOI:** 10.1038/sdata.2018.59

**Published:** 2018-04-10

**Authors:** Taylor H. Leach, Luke A. Winslow, Frank W. Acker, Jay A. Bloomfield, Charles W. Boylen, Paul A. Bukaveckas, Donald F. Charles, Robert A. Daniels, Charles T. Driscoll, Lawrence W. Eichler, Jeremy L. Farrell, Clara S. Funk, Christine A. Goodrich, Toby M. Michelena, Sandra A. Nierzwicki-Bauer, Karen M. Roy, William H. Shaw, James W. Sutherland, Mark W. Swinton, David A. Winkler, Kevin C. Rose

**Affiliations:** 1 Rensselaer Polytechnic Institute, Department of Biological Sciences, Troy, New York 12180, USA.; 2 Patrick Center for Environmental Research, Academy of Natural Sciences of Drexel University, Philadelphia, Pennsylvania 19103, USA.; 3 New York State Department of Environmental Conservation, Albany, New York 12233, USA.; 4 Darrin Fresh Water Institute, Bolton Landing, New York 12814, USA.; 5 Virginia Commonwealth University, Center for Environmental Studies and Department of Biology, Richmond, Virginia 23111, USA.; 6 New York State Museum, Cultural Education Center, Albany, New York 12230, USA.; 7 Department of Civil and Environmental Engineering, Syracuse University, Syracuse, New York 13244, USA.; 8 U.S. Environmental Protection Agency, Clean Air Markets Division, Washington, DC 20460, USA.; 9 Wenzhou-Kean University, Department of Biology, Wenzhou, Zhejiang Province 325060, China.; 10 New York State Department of Environmental Conservation, Division of Air Resources, Adirondack Long Term Monitoring Project, Ray Brook, New York 12944, USA.; 11 Sullivan County Community College, Science Division, Loch Sheldrake, New York 12759, USA.; 12 Marist College, Poughkeepsie, New York 12601, USA.; 13 Cary Institute of Ecosystem Studies, Millbrook, New York 12545, USA.

**Keywords:** Ecosystem ecology, Limnology, Climate-change impacts

## Abstract

Concurrent regional and global environmental changes are affecting freshwater ecosystems. Decadal-scale data on lake ecosystems that can describe processes affected by these changes are important as multiple stressors often interact to alter the trajectory of key ecological phenomena in complex ways. Due to the practical challenges associated with long-term data collections, the majority of existing long-term data sets focus on only a small number of lakes or few response variables. Here we present physical, chemical, and biological data from 28 lakes in the Adirondack Mountains of northern New York State. These data span the period from 1994–2012 and harmonize multiple open and as-yet unpublished data sources. The dataset creation is reproducible and transparent; R code and all original files used to create the dataset are provided in an appendix. This dataset will be useful for examining ecological change in lakes undergoing multiple stressors.

## Background & Summary

Freshwater lakes are changing in complex ways, with multiple long-term environmental stressors interacting to form novel conditions in aquatic ecosystems. Most lakes globally are undergoing temperature warming in response to climate change^1,2^. Many lakes also face concurrent stressors such as acidification and subsequent recovery^3–5^, browning^6^, eutrophication^7,8^, invasive species^9,10^, and/or increased extraction for drinking water or irrigation^11^. While some stressors act at global scales (e.g., climate change), many stressors are local or regional. For example, many lakes in the northeastern U.S. and northern Europe were strongly acidified in past decades due to sulfur and nitrogen deposition from emissions from fossil fuel combustion and agricultural activity^12,13^ and have begun recovering since then in response to regulated decreases in emissions^4,5,14^. In agricultural areas, nutrient use and consequent eutrophication continue to result in water-quality issues such as anoxia^15,16^ and harmful algal blooms^17^.

Long-term data are critical to understanding and predicting the effects of ecosystem stressors that may act on decadal to multi-decadal scales^18^. Moreover, ecosystems can experience multiple, concurrent stressors. Understanding the effects of multiple, concurrent stressors is a critical need as disturbance regimes may interact to alter the trajectory of important biological and biogeochemical phenomena in complex ways. Regional and global-scale changes that occur simultaneously highlight the critical need for quality, long-term data on lake ecosystems that describe processes, interactions, and responses to multiple stressors. A number of existing long-term limnological datasets have been used to understand some aspects of long-term ecosystem change. For example, a recent analysis of eleven diverse lakes in the North Temperate Lakes (NTL) Long-Term Ecological Research (LTER) site has shown seasonal heterogeneity of water temperature warming in response to regional climate change^19^. Long-term monitoring of lakes in Europe and the United States have observed changes in water clarity^20^ and warming surface temperatures^21^ and a recently published 80-year data record showed the influence of re-forestation on long-term browning of Swedish lakes^22^. Such long-term datasets have formed the foundation of our modern understanding of limnological change. However, due to the many challenges associated with long-term data collections, the majority of long-term data sets focus on only a small number of lakes or response variables, but rarely both.

**Figure 1:**
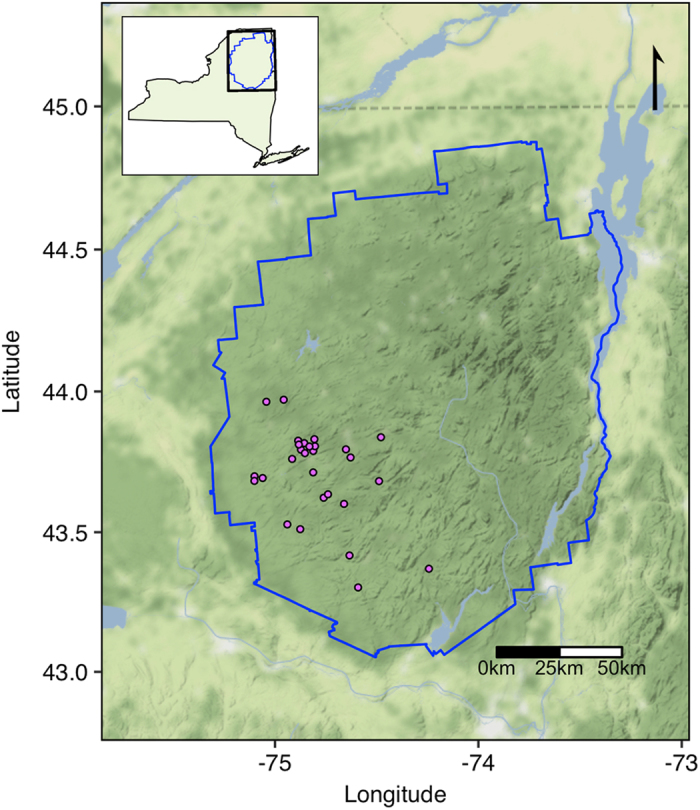
Location of study sites. The 28 study lakes (purple points) are located in the southwestern and south-central Adirondack Park (outlined in blue). Inset shows park location within New York, United States.

Here we present a 19-year database of physical, chemical, and biological data that span primary producers to secondary consumers measured during summers in 28 lakes in the Adirondack Park in New York State, USA (Fig. 1). The Adirondack Park is a protected state park in northeastern New York that encompasses c. 26,000 km^2^ of public and private land, and nearly 3000 lakes (> 0.4 ha)^23^. These lakes are poorly buffered due to surficial and bedrock geology, making them highly susceptible to acidification^24,25^. Due to the proximity to industrial centers in the mid-western US and prevailing winds^26^, the region received elevated atmospheric sulfur and nitrogen deposition, which has decreased in recent years^27^. This unique combination of geology and geography of the Adirondacks resulted in widespread and severe acidification of surface waters, which are now undergoing recovery. Concurrently, the northeastern U.S. has experienced substantial increases in temperature and precipitation and extreme events associated with changing climate^28^. These stressors individually may have contrasting impacts on aquatic ecosystems. For example, warming surface temperatures and increased thermal stability are predicted to decrease zooplankton species richness^29^, while recovery from acidification is associated with increases in zooplankton richness^30,31^.

The dataset presented here is a long-term, comprehensive record of physical, chemical, and biological measurements of a diverse set of lakes undergoing the effects of a changing climate while recovering from acidification. It is a harmonization of multiple open and unpublished data sources, including the Adirondack Effects Assessment Program (AEAP) Aquatic Biota Survey (www.rpi.edu/dept/DFWI/research/aeap/aeap_research.html), the Adirondack Long Term Monitoring Program (ALTM; www.adirondacklakessurvey.org), and the North American Land Data Assimilation System (NLDAS; http://ldas.gsfc.nasa.gov/nldas), and represents a more diverse, long-term data record of Adirondack lakes than has been previously available.

## Methods

### Site description

The 28 lakes in this dataset are located in the southwestern portion of the Adirondack Park in New York, USA (Fig. 1). This area received the highest rates of atmospheric deposition in the Adirondack Mountains^32^. When combined with inherently low acid neutralizing capacity (ANC)^24,25^, high rates of acidic deposition resulted in severe acidification of surface waters in this region^33,34^. The study lakes are located in five of the six major sub-drainage basins in the Adirondack region and span a range of size, depth, watershed area and hydrologic type (Table 1). The hydrologic classification scheme used was developed by (ref. 35) and is based upon a combination of hydrology (drainage or mounded seepage lakes), underlying geology (thickness of glacial till, or presence of calcite in the basin), and dissolved organic carbon (DOC) concentration (high or low), which combined characterize sensitivity to acidification of each lake. Of the 28 lakes, 20 are thin-till, drainage lakes, the class considered the most sensitive to acidification. Of these 20 thin-till drainage lakes, two have historically high DOC concentrations (TDH), while the remaining 18 have historically low DOC concentrations (TDL). There are six medium-till drainage lakes, two with historically high DOC concentrations (MDH) and four with historically low DOC concentrations (MDL). There is a single mounded seepage lake with historically low DOC (MSL) and one lake drains a watershed with deposits of carbonate (C), which eliminates sensitivity to acidification due to high ANC.

The lakes in this dataset were included in two independent long-term monitoring programs that were established to assess the effects of acid deposition in Adirondack lakes; the Adirondack Effects Assessment Program Aquatic Biota Study (hereafter referred to as AEAP) and the Adirondack Long Term Monitoring Program (hereafter referred to as ALTM). While both programs sampled more lakes than the 28 included in this dataset, these 28 lakes represent the overlap between the two separate programs and thus provide a comprehensive view of the long-term physical, chemical and biological characteristics of each lake. The data record starts in 1994 for all lakes and ends in 2006 for half of the lakes and in 2012 for the remaining half (Table 1). The physical, nutrient and biological data presented here were collected and analyzed by the AEAP. Additional water chemistry data were collected and analyzed as part of the on-going ALTM program. Because these monitoring programs were independent there is overlap in the measured water chemistry analytes. For analytes that were measured by both programs, we selected the data from a single program based upon completeness of record. Overlapping water chemistry measurements (i.e., those not selected from inclusion) can be found in the original data files (Data Citation 1; ‘data_inputs’) but not in the harmonized, final dataset presented here.

### Field collection methods

#### Sampling schedule (AEAP and ALTM)

As part of the AEAP, lakes were sampled three times during the summer (July, Aug, September) from 1994–1996. Starting in 1997, lakes were sampled twice per year (July and August). The ALTM program collected water chemistry data monthly, 12 months of the year starting in 1992 and is an on-going monitoring program (http://www.adirondacklakessurvey.org/). For the purposes of this paper the ALTM monthly chemistry data range from January 1994 to December 2012. For clarity of data sources we note the original program (AEAP or ALTM) that each data type in the subheadings below.

#### Physical characteristics (AEAP)

Temperature, dissolved oxygen (DO) and photosynthetically active radiation (PAR) measurements were taken at 1 m intervals throughout the entire water column in the deepest spot in each lake as part of the AEAP program. Temperature and DO were measured with a YSI Model 54 meter using a calibrated membrane electrode and thermistor (YSI, Yellow Springs, OH, USA). The thermocline depth was determined in the field as the depth at which the water temperature decreased≥2 **°**C in a meter. The thermocline depth determined the depths of epilimnetic samples for other variables (e.g., phytoplankton abundance and taxonomy). Secchi disk depth was also measured on each sampling occasion.

#### Chlorophyll and nutrient concentrations (AEAP)

Water samples to measure chlorophyll *a*, total nitrogen (TN), total phosphorus (TP), total filterable phosphorus (TFP), and molybdate reactive phosphorus (MRP) were collected at each study site coincident with the collection of the physical data (described above) and biological samples (see below). For sampling occasions when the water column was thermally stratified (as determined by the temperature profile) an integrated epilimnetic sample was collected with a 2.54-cm diameter hose. For un-stratified sampling events, a single integrated sample from the surface to 1 m above the bottom was collected. Samples were stored in high-density amber polyethylene bottles and transported in chilled coolers to the Keck Laboratory at Rensselaer Polytechnic Institute Troy, NY for processing and analysis^36,37^.

#### Water chemistry (ALTM)

The ALTM collected water samples for a suite of water chemistry parameters (Table 2). Samples were collected in two different ways depending on the mode of physical access and hydrology of the lake. For all lakes that were accessed by a helicopter and any lake without a surface outlet (see Table 1), samples were collected near the deepest part of the lake at 0.5 m below the surface with a Kemmerer sampler. For all other sites, water samples were collected at the lake outlet to allow safe sampling during periods of thin ice cover and because of limited helicopter availability. Samples were collected in high-density polyethylene bottles and transported in chilled coolers to the Adirondack Lakes Survey Corp. laboratory in Ray Brook, NY for processing and analysis^38^.

#### Phytoplankton (AEAP)

A single phytoplankton sample was collected in the deepest part of each lake from the surface down to the 1% PAR (estimated at twice the Secchi depth) with a 2.54-cm diameter integrated hose. For lakes shallower than the estimated 1% PAR depth, samples were collected from the surface to 1 m above the bottom. This approach contrasts with the methodology used for nutrients and chlorophyll *a*, which were collected as an integrated sample in the epilimnion. A 250-ml subsample of an integrated sample was preserved in the field with a 3% mixture of equal parts glutaraldehyde and formaldehyde for later enumeration and identification of species.

#### Zooplankton (AEAP)

Replicate zooplankton samples were collected in the deepest part of each lake from surface to 1 m above the bottom or to the depth where DO was<2 mg/L, whichever was shallower, using a hose-integration technique and constant-flow pump. The hose was lowered through the water column at a constant rate and at least 100 L were pumped from each lake (150–200 L for lakes identified as having low zooplankton densities) and concentrated with a 64- μm mesh. Zooplankton were narcotized with carbonated water and immediately preserved in the field with buffered formaldehyde.

### Sample Processing

#### Water chemistry (ALTM)

Aliquots of water samples were divided as necessary for the measurement of each analyte following standard methods outlined in Table 2 and briefly described here. Water color was determined on an unfiltered water sample by visual comparison to a platinum-cobalt standard. Conductivity, pH and ANC were measured electrometrically using a calibrated Orion or YSI glass electrode. Conductivity and pH where measured directly, with pH measured in the field immediately after collection, while ANC was measured using Gran titration^39^.

A Technicon Autoanalyzer (Seal Analytical, Inc., Mequon, Wisconsin, USA) was used for colorimetric determination of concentrations of ammonium (NH_4_^+^), reactive silica (SiO_2_), total monomeric aluminum (Al_TM_) and organic monomeric aluminum (Al_OM_) on an aliquot; the NH_4_^+^ samples were acidified with sulfuric acid prior to colorimetric analysis. Inorganic monomeric aluminum (Al_IM_) concentration was estimated as the difference between Al_TM_ and Al_OM_ (refs 40,41). Major anions including sulfate (SO_4_^2−^), nitrate (NO_3_^−^), fluoride (F^−^) and chloride (Cl^−^) concentrations were measured chromatographically^42,43^ with a Dionex ICS-1100 ion chromatograph (Thermo Fischer Scientific, Waltham, MA, USA). Base cations including sodium (Na^+^), potassium (K^+^), magnesium (Mg^2+^), and calcium (Ca^2+^) were measured with a PinAAcle 900H atomic absorption spectrophotometer (PerkinElmer, Waltham, MA, USA)^44^. Total dissolved aluminum (Al_TD_) was also measured with a PinAAcle 900H atomic absorption spectrophotometer but one fitted with a high-temperature graphite furnace and an AS900 auto sampler (PerkinElmer, Waltham, MA) to volatilize the inorganic and organic Al complexes^40,45^. A Tekmar Dorhmann Pheonix 8000 carbon analyzer (Teledyne Tekmar, Mason, OH, USA) was used to measure concentrations of dissolved organic and inorganic carbon (DOC and DIC, respectively) by converting the carbon in the sample to carbon dioxide and measuring the carbon dioxide with an infrared spectroscopic sensor^45^. A filtered aliquot (0.45 micron pore size GFF), preserved with phosphoric acid, was used to determine DOC via UV persulfate oxidation. DIC was measured in a separate sealed water sample collected in the field to ensure the DIC was not lost to the atmosphere and therefore underestimated^46^.

#### Chlorophyll and nutrient concentrations (AEAP)

As with the water chemistry, aliquots of water samples were divided as necessary and measured with standard methods outlined in Table 2 and described here. Chlorophyll *a* concentration was determined by filtering water sampled onto a glass fiber filter, extracting the chlorophyll *a* in 90% acetone for 4–24 h and measuring fluorescence with a Turner MODEL 10- AU fluorometer^47^ (Turner Designs, Sunnyvale, CA, USA).

Total nitrogen (TN) and total phosphorus (TP) concentrations were measured on a well-mixed unfiltered aliquot of lake water while total filterable phosphorus (TFP) was measured on filtrate passed through a 0.45-micron membrane filter. TN was measured using persulfate oxidation^48^. For TP and total filterable phosphorus (TFP) concentrations, aliquots were digested in a potassium persulfate solution via autoclave at high heat, then determined colorimetrically using a spectrophotometer^45^. Molybdate reactive P (MRP) and ammonium (NH_4_^+^) were measured on raw water samples. While this differs slightly from the standard methods, particulates are so low in these lakes that using unfiltered samples should have had little effect on the outcome. Both MRP and NH_4_^+^ were measured colorimetrically via flow injection (Lachat QuikChem Flow Injection Analysis System, Hach Company, Loveland, CO, USA)^45^. Note that NH_4_^+^ appears in both the nutrient and water chemistry data sets. The same procedure was used to estimate NH_4_^+^ concentration but the location and depth of the samples differed. The AEAP data set measure NH_4_^+^ concentration from an integrated epilimnetic sample near the deep spot while the ALTM measured NH_4_^+^ concentration at 0.5 m near the deep spot or at the lake outlet depending upon the lake (see Table 1 for details).

#### Phytoplankton (AEAP)

Phytoplankton samples from 1994 and 1995 were analyzed at the University of Louisville (Louisville, Kentucky, USA). All samples from 1996 or later were analyzed at the Patrick Center for Environmental Research at the Academy of Natural Sciences of Drexel University (Philadelphia, Pennsylvania, USA) hereafter referred to as ANS. At the University of Louisville, samples were filtered onto a membrane filter, cleared and mounted under a coverslip on a microscope slide^49^. One to three slides were prepared for each sample and 10–30 fields per slide were examined under 625x magnification. At ANS the samples were concentrated by centrifuge and examined under 538x magnification with an inverted microscope using Utermöhl sedimentation technique and counting random fields^50,51^. Approximately 500 natural units were enumerated for each sample. Identifications of phytoplankton were made to the species level when possible using keys^52–59^. All taxonomy was updated according to^60^ as of October 2017. All taxonomic information and updates are shown in the phytoplankton reformat table (Data Citation 1, ‘data_inputs’ folder).

To determine biovolumes of algal taxa a simple geometric shape was matched to an individual cell, 1 to 3 dimensions of the cell were measured and these measurements were used to calculate the volume (in μm^3^). Fifteen specimens were measured for each taxon with additional measurements for larger and variably sized taxa. In several cases of rare taxa, fewer specimens were measured and/or sizes were determined from literature values.

#### Zooplankton (AEAP)

Crustacean zooplankton were counted in a Bogorov chamber under 60x magnification using standard subsampling techniques with subsamples 1–5 ml in volume^61,62^. Rotifers were counted in a Sedgewick-Rafter cell (1 ml subsample) under 100x magnification. All individuals were identified to species when possible. Crustacean zooplankton were identified using keys from (refs 63–66), while rotifers were identified using^66–70^. All zooplankton counts and identifications were identified and counted under the supervision of W.H. Shaw with the exception of 2000–2002 when samples were counted at Marist College (Poughkeepsie, New York, USA) using standard methods. Taxonomy of the original dataset has been revised to reflect updated classification as of January 2017 based on (refs 71–73). Taxonomic updates and information are in separate ‘reformat tables’ for crustacean zooplankton and rotifers for ease of updating in the future (Data Citation 1, ‘data_inputs’ folder).

Zooplankton biomass was estimated from the count data using published empirical length-weight relationships (crustacean zooplankton) or formulas for body volume calculations (rotifers) for the freshwater zooplankton species in the dataset or for congeners when necessary. For the rotifers, body volume formulas are from (refs 74,75). Length-weight regressions for the crustacean zooplankton are from (refs 75–81).

Since size measurements were not taken of the zooplankton during the enumeration procedure, we used average organism lengths for each species from published studies or from the North Temperate Lakes Long-Term Ecological Research site (NTL LTER; https://lter.limnology.wisc.edu/data). The values derived from the NTL LTER are the average length of all individuals within a species collected across all seven lakes from 1982-2015 (year-round sampling). Measurements for an additional seven crustacean species are from (refs 76,77,82–84), or from a small glacial lake in the Poconos Mountains of Northeastern PA (*T. Leach unpublished data*). Measurements for rotifers are from (refs 74,75) or the North Temperate Lakes LTER zooplankton dataset 1982–2015 (http://lter.limnology.wisc.edu). See Data Citation 1, zoop_biomass_conversion.csv for all equations, measurements, and references.

Published length-weight relationships for the crustacean zooplankton typically incorporate dry weights of an individual. For consistency with rotifers and phytoplankton biomass estimates, we converted dry weights to wet weights by assuming that the ratio of dry:wet weight was 0.1(10%) following (ref. 85). For the phytoplankton and some of the rotifer species, the estimates are expressed as volume (mm^3^ individual^−1^). For comparison with the crustacean zooplankton biomass, biovolume was converted to biomass by assuming that all organisms had a density of 1 (ref. 85); from this assumption organism volume as μm^3^ individual^−1^ is equivalent to biomass as μg individual^−1^.

### Meteorological data

Long-term meteorological data, including air temperature, relative humidity, wind speed, and downwelling shortwave and longwave radiation, were extracted from the North American Land Data Assimilation System (NLDAS) from 1979–2012 using the geographic location of each lake. NLDAS is a gridded reanalysis of historic weather data over North America produced and maintained as a collaboration between NASA and NOAA (http://ldas.gsfc.nasa.gov/nldas). Meteorological data from NLDAS were averaged (simple mean) to represent daily values for each variable.

### Data harmonization

**Figure 2:**
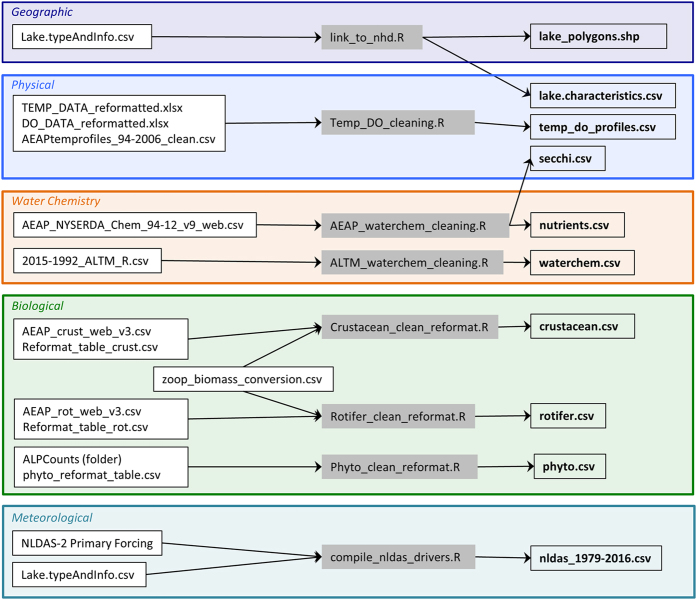
Workflow diagram for data cleaning and harmonization. Input files on the far left, R code scripts in the grey boxes and output files (.csv format) are on the right. Information on the output files can be found in Table 3. All original data files and scripts to re-create each file are available at Data Citation 1.

We harmonized the different data sources using a combination of lake names and latitude/ longitude records. We verified all lake names against the Geographical Names Information System database (https://nhd.usgs.gov/gnis.html) using latitude and longitude reference. Further, to connect the dataset with a physical water body, we linked each site with its corresponding polygon in the high-resolution U.S. Geological Survey’s National Hydrography Dataset (NHD) and include corresponding polygons and permanent identifiers for future use. Sampling date formats and lake names were also standardized so that data files can be easily linked by lake and sampling occasion in addition to permanent identifiers. See Fig. 2 for a detailed workflow and relationship between each data type.

### Code availability

All key harmonization and data conversion steps were done in the R scientific computing language version 3.3.3 (ref. 86). For reference, original data files and all harmonization R code are included in a Data Citation 1, ‘data_input’ and ‘Code_toclean’ folders, respectively.

## Data Records

The data are available in two formats; as comma separated files (.csv) within the folder ‘data’ (Data Citation 1) and as an R Data Package wrapper, *adklakedata* (Data Citation 2), which automatically retrieves and makes the data files available in the R programming environment^86^. Both the ‘data’ folder within Data Citation 1 and the *adklakedata* package contain the same data.

There are several different categories of data in the dataset: (1) geographic, (2) physical, (3) water chemistry, (4) biological, (5) meteorological and (6) other (Table 3, Fig. 2). Additionally, each.csv data file has an accompanying text file with the same name that contains a description of each column header, units of each variable and other pertinent metadata. Data are split across files containing different types of data based on data structure but all data files contain a column with the unique lake name and date on which the data were measured, which enables linking data files together for analysis (See data Usage from more information). A list with a description of the files associated with the dataset is provided in ‘adklake_data_descriptions.txt’ and Table 3. This information is also available in the *adklakedata* documentation available on CRAN, the Comprehensive R Archive Network (https://cran.r-project.org/).

## Technical Validation

There were two types of technical validation performed on these data. The first involved extensive quality assessment and quality control (QA/QC) of the data collection and sample analysis methods. The second included validation of the data cleaning and harmonization to create a unified and compatible data structure across all data types.

### Data collection and sample processing validation

A QA/QC program for the AEAP chlorophyll *a* and nutrient samples consisted of running a certified external standards every 10th sample, spikes in 10% of samples to verify analyte recovery and replication of sample analysis for 10% of samples. Standard curves were run at the beginning and end of every analyte analysis batch (20 samples) as well as a blank and standard in the middle of each run to assess drift and develop the standard curve. If the value of these standards was not within 10% of expected the entire batch was re-analyzed. The Keck Laboratory where the AEAP samples were analyzed was Environmental Laboratory Accreditation Program and National Environmental Laboratory Accreditation Conference certified and participated in the USGS Standard Water Sample Program administered by the Environment Canada, National Water Research Institute Ecosystem Inter-laboratory Quality Assurance Program. As part of this program, proficiency samples were analyzed every six months to assure quality control and the laboratory was audited every two years.

QA/QC procedures for all samples counted at the ANS included recounts of specific samples and consultation with outside experts for verification of taxonomy. Outside experts included Dr Ann St. Amand (PhytoTech, St. Joseph, Michigan, USA), Dr Rex. L. Lowe (Bowling Green State University, Bowling Green, Ohio, USA) and William R. Cody (Aquatic Taxonomy Specialists, Malinta, Ohio, USA). Dr Ann St. Amand also verified taxonomy of samples counted at the University of Louisville. Also, images were taken of most taxa to help insure consistency of identifications from the beginning to the end of the project, especially for undescribed taxa.

To ensure consistent species identification of the zooplankton, photographs were taken for both rotifers and crustaceans. When possible, microscope slides were created for crustaceans showing important anatomic criteria. Calanoid and cyclopoid species identifications were verified with prepared slide mounts of antenna and 5th leg preparations of both males and females when possible. When congeneric species or multiple species of *Daphnia* were present, slides of 25 randomly selected individuals were prepared to estimate the relative proportions of each congener and compared to proportions within full counts. Identification of reoccurring but rare rotifer species were verified by Dr Richard Stemberger (Dartmouth College, Hanover, NH). For all zooplankton samples the subsample-to-sample ratio was maximized in order to limit multiplication errors and improve accuracy of counts. Duplicates counts were performed on samples from every tenth lake during the 1994–1996 and 2001–2002 sampling periods. These duplicate counts showed that counting precision was high.

The water chemistry data collected as part of the ALTM sampling program also had a QA/QC program in place to assure data quality and measurement accuracy. This procedure included a clear line of sample custody, standard maximum holding times and assessment of analytical precision. To assess analytical precision, 5% of all samples were collected and analyzed in triplicate. On days when field triplicates were collected the values in the dataset represent the average of those triplicates. Laboratory duplicates (i.e., samples split in the laboratory from the same field collection container) were analyzed every 20th sample. Field blanks were also created for at least 5% of the total field samples. Field blanks were prepared in the laboratory by filling collection containers with deionized water and then processing them in the field as though they were field samples. Analytical standards were run at the beginning of a batch and every tenth sample. Only field samples bracketed by passing standards were accepted. Correction actions such as recalibration and sample re-runs were performed if coefficient of variation between laboratory duplicates or standards was greater than 0.1.

### Data harmonization validation

The R code to restructure, and harmonize the data, as well as the original data files are included in the folders called ‘Code_toclean’ and ‘input_data’ in Data Citation 1. All of the code was written by T. Leach and reviewed by L. Winslow. A series of manual QA/QC steps were performed to verify that there were no data processing errors between the raw source files and final data tables. A random 1% of each data type was manually checked between the original and final data files. All physical data including temperature and dissolved oxygen profiles and Secchi disk depths were manually checked for out of range or unexpected values. Out of range values were corrected or removed where appropriate. The database and R code were revised as needed throughout these manual validation steps to correct mistakes.

## Usage Notes

The combined dataset is distributed as a series of comma separated value (CSV) files that contain the data organized by data type (See Table 3 for description of each data type). Despite being separate files, all data can be linked by geographic location (site) using ‘lake.name’ or ‘PERMANENT_ID’ (from the NHD), or on a temporal axis using the ‘date’ variable. Keep in mind that not all chemical, physical and/or biological data were collected on the same day so a matching window (for example±7 days) may be useful to employ when merging different data types for analysis.

We have developed two methods for data access. One, the CSV files of all data can be downloaded directly from an online repository (Data Citation 1). This supports general use cases, as CSV is a common and widely supported data format. Two, we have developed an R package wrapper for the dataset that is available from CRAN, the Comprehensive R Archive Network. This package *adklakedata* automates the downloading, local storage, and access of the data. Data are accessed using the ‘*adk_data*’ function which accepts a parameter for each dataset (e.g., `*adk_data(‘tempdo’)*’ for temp and dissolved oxygen data).

## Additional information

**How to cite this article:** Leach, T. H. *et al.* Long-term dataset on aquatic responses to concurrent climate change and recovery from acidification. *Sci. Data* 5:180059 doi: 10.1038/sdata.2018.59 (2018).

**Publisher’s note:** Springer Nature remains neutral with regard to jurisdictional claims in published maps and institutional affiliations.

## Supplementary Material



## Figures and Tables

**Table 1:** Characteristics of 28 lakes in dataset.

**Lake**	**Lat.**	**Long.**	**Hydro. type**	**Max. depth (m)**	**Mean depth (m)**	**Lake volume (m**^**3**^ **x 10**^**3**^)	**Surface area (ha)**	**End date**
Big Moose	43.816874	−74.856111	TDL	21.3	6.8	34882	512.5	2012
Brooktrout*	43.600966	−74.660624	TDL	23.2	8.4	2420	28.7	2012
Carry*	43.682037	−74.488558	MSL	4.6	2.2	62	2.8	2006
Cascade	43.789104	−74.812042	MDL	6.1	4.2	1719	40.4	2012
Constable	43.831008	−74.806420	TDL	4.0	2.1	435	20.6	2006
Dart	43.793758	−74.872572	TDL	17.7	7.3	3807	51.8	2012
G*	43.417142	−74.633945	TDL	9.8	4.5	1437	32.2	2012
Grass*	43.693004	−75.060844	MDL	5.2	1.5	78	5.3	2006
Indian*	43.622864	−74.760748	TDL	10.7	3.0	981	33.2	2012
Jockeybush*	43.302775	−74.591444	TDL	11.3	4.5	786	17.3	2012
Limekiln	43.713005	−74.812459	TDL	21.9	6.1	11476	186.9	2012
Long	43.837892	−74.479025	TDH	4.0	2.0	33	1.7	2006
Loon Hollow*	43.963601	−75.042530	TDL	11.6	3.4	191	5.7	2006
Middle Branch*	43.699117	−75.100869	TDL	5.2	2.1	363	17.0	2006
Middle Settlement*	43.682807	−75.101427	TDL	11.0	3.4	545	15.8	2006
Moss	43.781396	−74.852986	MDL	15.2	5.7	2598	45.7	2012
North*	43.527752	−74.939567	TDL	17.7	5.7	10107	176.8	2012
Queer*	43.805956	−74.803521	TDL	21.3	10.9	5960	54.5	2006
Raquette	43.794924	−74.651303	MDH	3.0	1.6	24	1.5	2006
Rondaxe	43.760879	−74.915920	TDL	10.1	3.0	2733	90.5	2012
Sagamore	43.766050	−74.628371	MDH	22.9	10.5	7131	68	2012
South*	43.510956	−74.875888	TDL	18.3	8.3	16302	197.4	2012
Squash	43.825567	−74.886135	TDH	5.8	1.4	45	3.3	2006
Squaw*	43.635083	−74.739599	TDL	6.7	3.4	1249	36.4	2012
West	43.811890	−74.882960	TDL	5.2	1.5	152	10.4	2006
Willis	43.369628	−74.243171	MDL	2.7	1.6	229	14.6	2006
Willys*	43.970776	−74.957396	TDL	13.7	4.9	1188	24.3	2006
Windfall	43.804966	−74.830768	C	6.1	3.2	78	2.4	2006
Geographic coordinates identify the lake, not necessarily the exact sampling location. End date refers to last year that all data types are available; all data start in 1994. Note that water chemistry extends to 2012 for all lakes. First two letters of the abbreviations for hydrologic type (hydro. type) are: TD=thin till, drainage; MD=medium till, drainage; MS=mounded, seepage. The last letter refers to historical DOC concentration (L=low (< 500 μM) or H=high>500 μM). Windfall is a carbonate lake (C). Information compiled from (ref. 87) and (ref. 36).								

*water chemistry data typically collected by helicopter near the deep spot of the lake.

**Table 2:** Analytical methods used for all analytes in the database.

**Variable**	**Analytical Method**	**Reference**
Acid neutralizing capacity (ANC)	Gran titration	US EPA Method 310.1 (ref. 39)
Aluminum, total dissolved (Al_TD_)	Atomic absorption spectrophotometry with high-temperature graphite furnace	US EPA Method 200.9 (ref. 45)
Aluminum, total monomeric (Al_TM_)Aluminum, organic monomeric (Al_OM_)	Atomic absorption spectrophotometry	McAvoy *et al.* 1992 (ref. 41)
Aluminum, inorganic monomeric (Al_IM_)	Calculated as Al_TM_ - Al_OM_	McAvoy *et al.* 1992 (ref. 41)
Ammonium (NH_4_^+^)	Colorimetric	US EPA Method 350.1 (ref. 45)
Calcium (Ca^2+^)	Atomic absorption spectrophotometry	US EPA Method 215.1 (ref. 45)
Chloride (Cl^−^)Fluoride (F^−^)Nitrate (NO_3_^−^)Sulfate (SO_4_^2−^ )	Chromatography	US EPA Method 300.0 (ref. 43)
Conductivity	Electrometric	US EPA Method 120.1 (ref. 45)
Chlorophyll *a* (Chl)	Fluorometric	Turner (1985) (ref. 47)
Dissolved oxygen (DO)	Membrane electrode	US EPA Method 360.1 (ref. 45)
Dissolved inorganic carbon (DIC)Dissolved organic carbon (DOC)	UV/Persulfate Oxidation	US EPA Method 415.1 (ref. 45)
Magnesium (Mg^2+^)	Atomic absorption spectrophotometry	US EPA Method 258.1 (ref. 45)
Nitrogen, total (TN)	Persulfate oxidation	Langer & Hendrix (1982) (ref. 48)
pH	Electrometric	EPA AERP 05 (ref. 39)
Phosphorus, orthophosphate (MRP)	Colorimetric	US EPA Method 365.1 (ref. 45)
Phosphorus, total (TP)Phosphorus, total filterable (TFP)	Colorimetric	US EPA Method 365.4 (ref. 45)
Potassium (K^+^)	Atomic absorption spectrophotometry	US EPA Method 242.1 (ref. 45)
Silica (SiO_2_)	Colorimetric	US EPA Method 370.1 (ref. 45)
Sodium (Na^+^)	Atomic absorption spectrophotometry	US EPA Method 273.1 (ref. 45)
Water color	Colorimetric platinum (Pt-Co units)	US EPA Method 110.2 (ref. 45)

**Table 3:** Description of all distributed data files.

**File Name**	**Metdata file name**	**Description**
*Geographic*		
lake_polygons.shp	lake_polygons.txt	Shape file containing the polygon of all 28 lakes from the National Hydrography Dataset (high-resolution)
*Physical*		
lake_characteristics.csv	lake_characteristics.txt	Geographical location and physical characteristics of all 28 lakes in the dataset (include lake surface area, watershed area, hydrologic type, max and mean depth etc.) NHD identification numbers.
temp_do_profiles.csv	temp_do_profiles.txt	Water temperature and dissolved oxygen (profiles for each sampling event at 1 m depth intervals. The temperature data is resolved to 0.1 **°**C and the DO to 0.1 mg/L.
secchi.csv	secchi.txt	Secchi disk measurement for each sampling event, resolved to 0.1 m.
*Water Chemistry*		
waterchem.csv	waterchem.txt	Surface water chemistry parameters for each sampling event.
nutrients.csv	nutrients.txt	Nutrient and chlorophyll *a* concentration data for each sampling event.
*Biological*		
phyto.csv	phyto.txt	Cell counts and biovolumes for each sampling event. Typically identified to species.
rotifer.csv	rotifer.txt	Organisms L^−1^ for each sampling event. Typically identified to species.
crustacean.csv	crustacean.txt	Organisms L^−1^ for each sampling event. Typically identified to species but always to genus.
*Meteorological*		
nldas_1979-2016.csv	nldas_1979-2016.txt	Local meteorology for each lake subset from the North American Land Data Assimilation dataset averaged to a daily interval.
*Other*		
adklake_data_descriptions.txt	---	List with descriptions of each file in the dataset.
zoop_biomass_conversion.csv	zoop_biomass_conversion.txt	Formula and body size measurements used to convert organism count data to biomass. Includes references for formula, coefficients and measurements.
All data can be linked by a standardized lake.name and/or date. Metadata files contain information pertaining to the associated data file such as descriptions of column names, units, depths sampled etc.		
